# Differential Diagnosis of Solitary Pulmonary Inflammatory Lesions and Peripheral Lung Cancers with Contrast-enhanced Computed Tomography

**DOI:** 10.6061/clinics/2016(10)01

**Published:** 2016-10

**Authors:** Zhi-gang Chu, Bo Sheng, Meng-qi Liu, Fa-jin Lv, Qi Li, Yu Ouyang

**Affiliations:** The First Affiliated Hospital of Chongqing Medical University, Department of Radiology, Chongqing, China

**Keywords:** Lung Diseases, Inflammation, Lung Cancer, Computed Tomography

## Abstract

**OBJECTIVES:**

To clarify differences between solitary pulmonary inflammatory lesions and peripheral lung cancers with contrast-enhanced computed tomography.

**METHODS:**

In total, 64 and 132 patients with solitary pulmonary inflammatory masses/nodules and peripheral lung cancers, respectively, were enrolled in this study. Their computed tomographic findings were summarized and compared retrospectively.

**RESULTS:**

Compared with the peripheral lung cancers, the inflammatory lesions were located closer to the pleura (*p*<0.0001). The majority of the inflammatory lesions were patchy and oval-shaped (82.8%), whereas most of the tumors were lobulated (82.6%). Almost all the inflammatory cases were unclear (93.8%), whereas most of the tumors had spiculated margins (72.7%). Computed tomography values were significantly higher for the inflammatory lesions than for the cancers (*p*<0.0001). More than half of the inflammatory lesions had defined necrosis (59.3%). Furthermore, 49.2% of the cancers enhanced inhomogeneously, but only 24.6% had ill-defined necrosis or cavities. The peripheral zones of 98.4% of the inflammatory lesions and 72.7% of the tumors were unclear, with peripheral scattered patches (92.2%) and beam-shaped opacity (66.7%) being the most common findings, respectively. Adjacent pleural thickening was more frequent for the inflammatory lesions than the cancers (95.3% *vs*. 21.1%, *p*<0.0001), whereas pleural indentation was found in 67.4% of the subjects with cancer. In addition, hilar (*p*=0.034) and mediastinal (*p*=0.003) lymphadenopathy were more commonly detected in the cancers than in the inflammatory cases.

**CONCLUSIONS:**

Contrast-enhanced computed tomography findings for pulmonary inflammatory lesions and peripheral lung cancers were significantly different in many aspects. Developing a comprehensive understanding of these differences is helpful for directing their management.

## INTRODUCTION

Focal pulmonary lesions, which can be nodules or masses, are commonly encountered in clinical practice 1 and can be benign or cancerous. Common diagnostic methods for evaluating such lesions include sputum cytology, bronchoscopy, and transthoracic needle biopsy, depending on lesion location 1. In addition to these examinations, noninvasive procedures, especially computed tomography (CT), are also commonly applied to localize lesions and provide essential diagnostic information.

Inflammation and lung cancer are the most common types of pulmonary benign and malignant lesions, respectively. On chest CT scanning, most solitary pulmonary inflammatory lesions are found in the lung fields adjacent to the pleura. Peripheral lung cancers, predominantly adenocarcinoma and squamous cell carcinoma, are also usually located in these zones. These two lesion types typically exhibit similar CT manifestations; however, the procedures used to treat them are substantially different. Therefore, it is necessary to carefully distinguish these lesion types based on their CT characteristics.

Isolated pulmonary inflammatory lesions have an unknown etiology, but they are usually associated with a focal, uncontrolled inflammatory response to infection with bacteria, viruses or other organisms. The pathological course of this type of lesion mainly includes accumulation of multiple types of inflammatory cells as well as alteration and exudation, whereas that for lung cancer lesions includes abnormal cell proliferation and invasion into surrounding tissue. Focal inflammation can easily result in a peripheral response that includes exudation and edema. Meanwhile, infiltration and disruption of mesenchyme usually occur during tumor growth and these processes further affect surrounding structures 2-4. These pathological differences suggest that observable differences in CT manifestations may also exist.

Several previous studies have focused on differentiating benign and malignant pulmonary nodules (≤3 cm) with CT through evaluations of morphology, perfusion or growth rate 2,5-10. However, in all of the referenced studies, the sample size for benign nodules was very small and their pathological nature was extremely diverse. Furthermore, although inflammatory lesions were studied, their CT characteristics remained poorly understood. To date, no relevant study has comprehensively distinguished solitary inflammatory lesions from peripheral lung cancers using CT. Therefore, the aim of the current study was to clarify the CT characteristics of these two common conditions by comprehensively evaluating representative lesions and their surroundings. The provided information may be helpful for directing further management of these lesions.

## MATERIALS AND METHODS

### Patients

From 1 July 2012 to 31 December 2014, we collected CT data on patients with solitary pulmonary nodules or masses who consecutively underwent surgical resection (140 cases), bronchoscopy (76 cases), or transthoracic needle biopsy (37 cases) in departments of thoracic surgery and respiratory medicine. The authors had access to identifying information after data collection. Patients with confirmed solitary inflammatory lesions or peripheral lung cancers and those who had complete clinical and contrasted-enhanced CT data were included in this study. Lesions that were too large to generate adequate descriptions of their surrounding structures were excluded (3 cases). In addition, pathologically confirmed solitary tuberculosis (5 cases), fungal infection (3 cases) and noninfectious lesions (2 cases) were not evaluated in this study because of small sample sizes and the differences in their associated pathological processes. Moreover, pulmonary inflammatory pseudotumors were also excluded because they usually had distinct characteristics, appearing as asymptomatic, solitary, peripheral, sharply circumscribed masses with heterogeneous CT attenuation 11. No patients were excluded due to lack of information. In total, 64 patients with solitary inflammatory lesions and 132 with peripheral lung cancers were enrolled in this study. The collected clinical data included age, sex, smoking history, and clinical manifestations. Laboratory results included routine blood examinations for the patients with inflammatory lesions.

### CT Protocol

All patients were examined with a 64-multidetector CT scanner (LightSpeed VCT; GE Healthcare, Waukesha, WI, USA) with the following parameters: 120 kVp; 250 mAs; rotation time, 0.6 s; pitch, 0.984; collimation, 0.625 mm; slice thickness and interval for axial images, 5 mm/5 mm. The patients were placed in a supine position with both hands placed near the head when they underwent CT examination. Acquisition was performed from the level of the thoracic inlet to immediately inferior to the costophrenic angle. All patients underwent plain and enhancement CT scanning with a total of 80–100 mL (1.35 mL per kilogram of body weight) of nonionic iodinated contrast material ([Iopamidol], 320 mg/mL; Shanghai Bracco Sine Pharmaceutical Co., Ltd., China) at an injection rate of 3.0 mL/s, followed by 50 mL of saline solution via a power injector. Images were obtained with mediastinal (width, 350–450 HU; level, 20–40 HU) and lung (width, 1200–1600 HU; level, −500 to −700 HU) window settings.

### Image Analysis

All patients’ CT data were reviewed on a workstation (Advantage Workstation 4.6; GE Healthcare) by two senior chest radiologists who were blinded to the final results of the lesions. Interpretation discrepancy, if any, was resolved by consensus.

**Figure 1:**
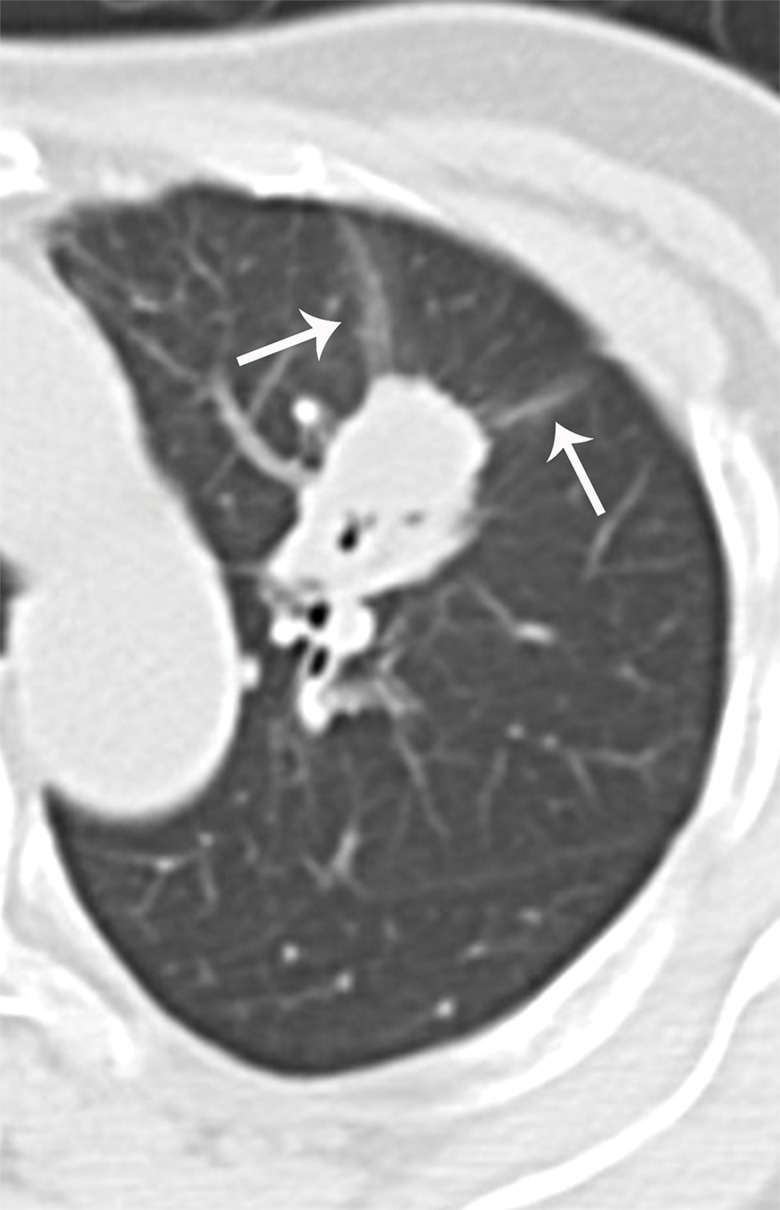
Beam-shaped opacity. Beam-shaped opacity refers to the shadow with ground glass opacity located at the side of a tumor close to the pleura. It is usually curved and has different directions, similarly to a light beam (arrows).

The numbers of lesions in different lobes (left superior and inferior lobes; right superior, middle, and inferior lobes) were counted. The locations of lesions were described according to the distance between them and the adjacent pleura. For these locations, two conditions were considered: space *vs*. no space. If space was observed, the distance between the lesions and the pleura was measured. Lesion shape was described as patchy (rectangular, triangular, or trapezoidal), oval, round, lobulated, or irregular. Lobulated lesions had an undulating contour, while irregular ones had an uneven shape that could not be characterized as patchy, oval, round, or lobulated. Lesion margins were described as unclear, smooth, or spiculated. Lesion size was calculated as half of the sum of the long and short transverse diameters. CT manifestations in the peripheral zones of the lesions were described as follows: scattered patches, beam-shaped opacity (Figure 1), nodules, fibrosis, halo signs and consolidation. Density and CT values were measured on plain and enhanced CT images. The enhanced CT value (ΔCT) was calculated to describe the degree of enhancement. On enhanced CT images, we determined whether necrosis was present in the lesions. The changes to the pleura adjacent to the lesions included significant, mild, or zero thickening and pleural indentation. Pleural effusions and lymph nodes in the hilum and mediastinum were also evaluated. Enlarged mediastinal and hilar lymph nodes were generally defined on chest CT scans as those with a diameter >1 cm in the short axis 12,13.

### Statistical Analysis

Clinical data, laboratory results, and various CT features were statistically analyzed for each patient. Continuous variables were expressed as the mean±SD, whereas categorical variables were expressed as numbers and percentages. A chi-square test was used to compare the frequencies of different clinical symptoms and CT features between inflammatory lesions and peripheral lung cancers. Independent sample *t*-tests were used to compare age; smoking index; and size, density and ΔCT of lesions between patients with inflammatory lesions and those with peripheral lung cancers. To determine the effect of ΔCT on differentiating inflammatory from neoplastic lesions, receiver operating characteristic (ROC) analysis was performed. All analyses were performed with the statistical package SPSS (version 18.0 for Windows, SPSS Inc., Chicago, Illinois, USA). Two-tailed *p-*values of <0.05 were considered statistically significant.

## RESULTS

### Study Population

All the patients’ baseline clinical data and laboratory results are summarized in Table 1. The patients with lung cancers were older than those with inflammatory lesions (*p*=0.003). Compared with the lung cancer group, there were more male patients (84.3% *vs*. 65.1%, *p*=0.005) and more cases with respiratory symptoms such as cough, expectoration, phlegm with blood, and chest pain (93.8% *vs*. 49.2%, *p*=0.005) in the inflammation group.

### Pathologic

Pathologically, the peripheral lung cancer cases included 97 (73.5%) adenocarcinomas, 31 (23.5%) squamous carcinomas, three (2.3%) adenosquamous carcinomas and one (0.7%) mucoepidermoid carcinoma. As for the inflammatory lesions, the excised specimens were usually irregular in shape, grayish white in color, and had no clear boundary with adjacent lung tissue. Additionally, the texture of the lesions was rubbery. The terminal bronchioles in the masses proliferated and dilated, and multinucleated giant cells and neutrophils could be observed. Plasma cells and lymphocytes were distributed around bronchial walls. Alveolar epithelium actively proliferated, which resulted in focal pulmonary carnification. Large numbers of foam cells were detected in the alveolar spaces. Fibrous tissue proliferation with hyaline and myxoid degeneration occurred in the alveolar septa. Additionally, localized abscess formation could be detected in some lesions.

### Lesion Locations

**Figure 2:**
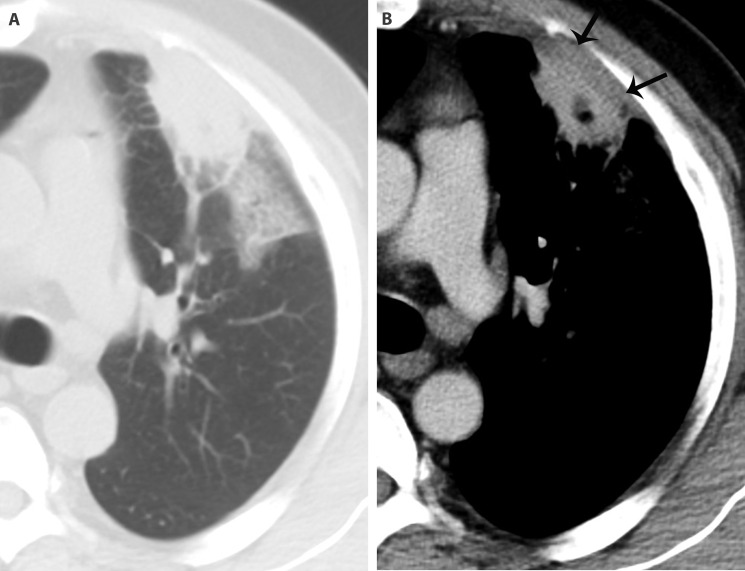
An inflammatory patch in a 48-year-old man with cough, expectoration and chest pain. There is a subpleural patch in the superior lobe of the left lung. Its interface is ill-defined, and ground glass opacity can be detected in the peripheral lung field (A). The basement of the lesion is broad. On the enhanced image, there is a low-density area with a clear boundary in it, which indicates necrosis. Significant pleural thickening (arrows) can be seen (B).

The locations of the inflammatory lesions and lung cancers are summarized in Table 2. The numbers of inflammatory lesions distributed in the superior and inferior lobes of the lungs were the same. In contrast, the cancers were more frequently found in the two superior lobes. Considering the distance between the lesions and the pleura, the inflammatory cases were more often found close to the pleura than were the cancers (*p*<0.0001). For the lesions in close proximity to the pleura, the inflammatory lesions usually had a broad basement (Figure 2), while acute angles between the cancerous nodules and the pleura were generally observed.

### CT Characteristics of Lesions

**Figure 3:**
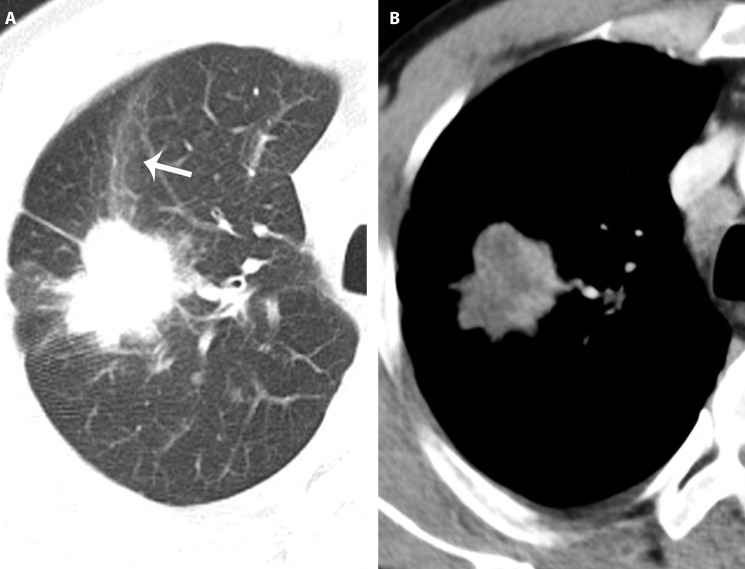
A peripheral pulmonary adenocarcinoma in a 49-year-old man with cough. A lobulated mass with spiculated margin located in the superior lobe of right lung. Beam-shaped opacity (arrow) can be detected in the peripheral lung field (A). On the enhanced image, an irregular low-density area with no clear boundary is seen (B).

The CT characteristics of the solitary inflammatory lesions and the peripheral lung cancers are summarized in Table 3. The majority of the inflammatory lesions were patchy and oval-shaped (53 cases, 82.8%). In contrast, most of the lung cancers were lobulated (109 cases, 82.6%). Considering their interfaces, 60 (93.8%) inflammatory cases were unclear and 96 (72.7%) cancers were spiculated (Figures 2 and 3). The densities of the inflammatory and carcinoid lesions measured with plain CT were similar (*p*=0.088); however, ΔCT values were significantly higher for the inflammatory lesions than for the cancers (*p*<0.0001). The diagnostic performance of ΔCT in diagnosing inflammatory lesions and peripheral lung cancers, expressed as an area under the curve (AUC), was 0.765 (95% CI: 0.699, 0.822; *p<*0.0001) with a cutoff of 40 HU. The sensitivity and specificity for inflammatory lesions were 81.3% and 64.4%, while those for peripheral lung cancers were 64.4% and 81.3%, respectively. On enhanced images, more than half of the inflammatory cases exhibited necrosis (38 cases, 59.3%), which usually manifested as a rounded and clear low-density area without enhancement (Figure 2), and 16 of these cases (42.1%) had pneumatosis. For the lung cancer cases, 65 cases (49.2%) enhanced inhomogeneously (Figure 3), and 16 of these cases (24.6%) had ill-defined necrosis or cavities. Necrosis was more commonly detected in the inflammatory lesions than in the cancers (*p*<0.0001).

### Lesion Surroundings

The peripheral lung field of 63 (98.4%) of the inflammatory lesions was unclear. Scattered patches, small nodules, or fibrosis could be detected. Beam-shaped opacity, small patches and halo signs were common manifestations around the cancers. Scattered patches (59 cases, 92.2%) and beam-shaped opacity (88 cases, 66.7%) were the most common signs observed in the peripheral zones of the inflammatory lesions and cancers, respectively (Figures 2 and 3). Furthermore, beam-shaped opacity was more common in adenocarcinomas and cancers with spiculation than in other pathological types and cases with smooth margins [74.2% (72 cases) *vs*. 45.7% (16 cases); 86.5% (83 cases) *vs*. 13.9% (5 cases); both *p*<0.0001] (Figure 3). The number of cases with pleural thickening in the patients with inflammatory and carcinoid lesions were 61 (95.3%) and 23 (17.4%), respectively, and this difference was significant (*p*<0.0001). Additionally, significant pleural thickening was more common in the patients with inflammatory lesions (*p*<0.0001) (Figure 2). Pleural indentation was found in 89 (67.4%) subjects with cancer, whereas the patients with inflammatory lesions did not exhibit this characteristic.

### Pleural Cavities and Lymph Nodes in the Hilum and Mediastinum

Hilar and mediastinal lymphadenopathy was more common in the subjects with lung cancer than in those with inflammatory lesions (hilar: 39.4% *vs*. 18.7%, *p*=0.034; mediastinal: 32.5% *vs*. 12.5%, *p*=0.003). Pleural effusion was rare in both inflammatory lesions and lung cancers, with only three (4.7%) and nine (6.8%) patients exhibiting this characteristic, respectively.

## DISCUSSION

In the present study, a comparison of isolated inflammatory lesions and peripheral lung cancers indicated many significant differences in their corresponding CT findings. These CT characteristics reflected their different pathological bases and could be used to differentiate the lesions from each other.

Theoretically, pulmonary inflammatory lesions and peripheral lung cancers can occur at any lobe site; however, the present results showed that they had distinct distribution tendencies. Another study of small solitary pulmonary nodules detected during population-based CT screening for lung cancer showed similar results 14. This finding may have arisen because of the developmental differences associated with the two lesion types. For inflammatory lesions, the exudate usually begins in the distal air spaces adjacent to the visceral pleura; in contrast, peripheral lung cancers usually derive from distal bronchia. Thus, the gaps between the tumors and the pleura would show differences at early stages, until the lesions grew large enough to involve the chest wall 15. For subpleural lesions with pleural thickening, the lung cancers usually caused mild thickening due to focal invasion, whereas the inflammatory lesions usually resulted in obvious thickening. These differences may be helpful for differentiating the two conditions.

The inflammatory lesions usually did not have smooth margins or clean peripheral lung fields due to the inflammatory process 16. In contrast, the lung cancers usually had a lobulated appearance with spiculated margins. These differences can be attributed to the different growth rates of cancerous cells with different differentiations, the contraction of inner fibrosis, and the growth of tumor cells along the pulmonary interstitium 2-4,16-18. For some of the peripheral lung cancers in this study, pleural indentation was commonly detected, which was usually caused by the contraction of fibrous tissue inside the lesions 2. However, this characteristic was not specific because similar findings, including local pleural adhesion and the presence of pleural tags, can also be detected in small benign lung nodules 14,19. In benign lesions, these features are probably caused by a local process associated with inflammation. Thus, other characteristics of the lesions should be considered for discriminating lung nodules with such manifestations.

In the present study, an interesting CT feature was revealed for peripheral lung cancers, namely, the presence of beam-shaped opacity, which has not been described previously. This characteristic was commonly detected in adenocarcinomas, especially those with spiculation. This finding may be a consequence of tumor mesenchyme contraction resulting in corresponding alveoli collapse, with the decrease of gas in the alveolar space increasing the local attenuation. However, further research is needed to understand the pathological nature of this novel CT feature and to determine whether it is specific to lung cancer.

On contrast-enhanced images, the inflammatory lesions usually showed a higher peak enhancement than the lung cancers, and clear necrosis was more commonly detected in them. Previous studies have also confirmed this finding 10,20. The likely explanation for these discrepancies is that active inflammatory responses usually result in dilatation of local vessels and central liquefaction necrosis, while malignant tumor growth is a relatively slow process that involves neovascularization, in which degeneration is common but significant necrosis is rare. ROC curve analysis showed that peripheral lesions with ΔCT values greater than 40 HU were more likely to be inflammatory lesions. However, for lesions with ΔCT values lower than 40 HU, especially those with slight and no enhancement, benign lung nodules, such as tumors, chronic inflammation, and inactive tuberculomas, should be considered 21,22.

Thoracic lymphadenopathy may be another useful characteristic for differentiating the two conditions studied here. Focal inflammatory lesions usually exhibited only hilar lymphadenopathy, while both hilar and mediastinal lymphadenopathy were usually detected in lung cancers due to metastasis. In addition, patients' clinical symptoms should also be considered, although they may not be specific for any disease. In the present study, a greater number of patients with inflammatory lesions had chest symptoms than those with lung cancer. A previous study indicated that, although approximately 10% of lung cancers are detected in asymptomatic patients on chest radiography, most patients are symptomatic when diagnosed 23. These symptoms may be related to metastasis. Thus, for patients without chest symptoms, a careful determination of clinical history may provide useful diagnostic information.

In addition to CT examination, dynamic contrast-enhanced magnetic resonance (MRI) with quantitative diffusion-weighted imaging has also been used to determine whether solitary pulmonary lesions are benign or malignant 24. In addition, 2-[F-18] fluoro-2-deoxy-D-glucose-positron emission tomography (FDG-PET) is considered a superior predictor of malignancy in solitary pulmonary nodules (≤3 cm) without cavities or calcification 25. These noninvasive imaging methods can provide additional data for discriminating pulmonary benign and malignant lesions.

There were several limitations in the current study. First, large or extensive lesions were not studied because they could be easily distinguished and their surrounding features are difficult to evaluate. In contrast, differentiating smaller lesions is usually difficult in clinical practice. Second, we only reported the number of patients with lung cancers who had hilar or mediastinal lymphadenopathy, whereas whether they had metastasis was not clear because not all lymph nodes were resected. Indeed, lymphadenopathy was only a factor used for differentiating between lesions and whether metastasis was present was not important for the results. Third, all of the aforementioned characteristics were not sufficient for differentiating extremely small lesions. For these cases, CT-guided percutaneous transthoracic biopsy may be more useful. Finally, diagnostic imaging still should not replace pathological examination because none of the summarized CT features for discriminating between lesion types were specific.

In conclusion, solitary pulmonary inflammatory lesions and peripheral lung cancers have several distinct CT features that could be helpful in their differentiation. Subpleural irregular patchy or oval-shaped lesions with defined necrosis, unclear margins and peripheral lung fields as well as significant enhancement and pleural thickening were usually inflammatory lesions, while lobulated lesions distant from the pleura with inhomogeneous enhancement, spiculated interfaces, pleural indentation, and beam-shaped opacity were usually lung cancer. Understanding these differences is helpful for initially determining lesion type and then selecting whether early intervention should involve invasive examination, surgery or anti-inflammatory treatment and to support further observations with serial CT.

## AUTHOR CONTRIBUTIONS

Chu ZG, Ouyang Y and Sheng B conceived and designed the study. Chu ZG, Lv FJ, Li Q and Liu MQ collected and assembled data. Chu ZG, Sheng B and Liu MQ performed data analysis and interpretation. Chu ZG and Ouyang Y contributed to manuscript writing. All authors read and approved the final version of the manuscript.

## Figures and Tables

**Table 1:** Patients’ characteristics and laboratory results.

	Patients with IL (n=64)	Patients with PLC (n=132)	*p*-value
Age (years)	56±11	61±9	0.003
Men/women	54/10	86/46	0.005
Smokers	39 (60.9%)	81 (61.3%)	0.954
Smoking index	570±375	973±1746	0.050
Fever	14 (21.9%)	N	-
Cough	48 (75%)	51 (38.6%)	0.000
Expectoration	40 (62.5%)	32 (24.2%)	0.000
Phlegm with blood	26 (40.6%)	10 (7.6%)	0.000
Chest pain	19 (29.7%)	15 (11.3%)	0.001
Hemoptysis	2 (3.1%)	5 (3.7%)	0.815
No symptoms	4 (6.2%)	67 (50.8%)	0.000
WBC count (×10^9^)	7.3±2.5	N	-

Note: IL = Inflammatory lesion; PLC = Peripheral lung cancer; WBC= White blood cell; N = Not mentioned.

**Table 2:** Locations of the inflammatory masses and lung cancers.

		Patients with IL (n=64)	Patients with PLC (n=132)	*p*-value
Right lung	SL	17 (26.6%)	38 (28.7%)	-
	ML	2 (3.1%)	15 (11.3%)	-
	IL	14 (21.9%)	21 (15.9%)	-
Left lung	SL	14 (21.9%)	35 (26.5%)	-
	IL	17 (26.6%)	23 (17.4%)	-
Distance to the pleura	Yes	4 (6.2%)(1.2-2.7 cm)	89 (67.4%)(0.2-3.6 cm)	0.000
	No	60 (93.8%)	43 (32.6%)	0.000

SL= Superior lobe; ML= Middle lobe; IL = Inferior lobe.

**Table 3:** Characteristics of the inflammatory lesions and peripheral lung cancers.

		Patients with IM (n=64)	Patients with PLC (n=132)	*p*-value
Shape	Patchy	30 (46.9%)	-	-
	Irregular	4 (6.3%)	-	-
	Lobulated	-	109 (82.6%)	-
	Oval	23 (35.9%)	10 (7.6%)	0.000
	Round	7 (10.9%)	13 (9.8%)	0.813
Margins	Unclear	60 (93.8%)	-	-
	Smooth	4 (6.2%)	36 (27.3%)	0.000
	Spiculation	N	96 (72.7%)	
Size	(LD + SD)/2(cm)	3.3±1.2 (1.1-6.2)	3.2±1.1 (1.2-7.2)	0.509
Density	Plain CT value (HU)	30.9±12.6	27.0±15.7	0.088
	Δ CT value (HU)	51.1±14.8	36.4±16.0	0.000
	Necrosis	38 (59.3%)	16 (12.1%)	0.000
	Pneumatosis	16 (42.1%)	N	
Pleural thickening	Significant	33 (51.6%)	4 (3.0%)	0.000
	Mild	28 (43.7%)	19 (14.4%)	0.000
	No	3 (4.7%)	109 (82.6%)	0.000

LD = Long diameter; SD = Short diameter; Δ CT= Peak CT value on contrast enhanced CT scan – CT value on plain CT scan; N = None; - = Not applicable.
